# Unconventional Role of Caspase-6 in Spinal Microglia Activation and Chronic Pain

**DOI:** 10.1155/2017/9383184

**Published:** 2017-02-07

**Authors:** Temugin Berta, Jee Eun Lee, Chul-Kyu Park

**Affiliations:** ^1^Pain Research Center, Department of Anesthesiology, University of Cincinnati Medical Center, Cincinnati, OH, USA; ^2^Department of Physiology, College of Medicine, Gachon University, Incheon 21999, Republic of Korea

## Abstract

Chronic pain affects ~20% of the worldwide population. The clinical management of chronic pain is mostly palliative and results in limited success. Current treatments mostly target the symptoms or neuronal signaling of chronic pain. It has been increasingly recognized that glial cells, such as microglia, and inflammatory signaling play a major role in the pathogenesis of chronic pain. Caspases (CASPs) are a family of protease enzymes involved in apoptosis and inflammation. They are pivotal components in a variety of neurological diseases. However, little is known about the role of CASPs in microglial modulation as to chronic pain. In particular, our recent studies have shown that CASP6 regulates chronic pain via microglial inflammatory signaling. Inhibition of microglia and CASP signaling might provide a new strategy for the prevention and treatment of chronic pain.

## 1. Introduction

Pain is defined as an unpleasant sensory and emotional experience associated with actual or potential tissue damage. Acute pain is transient and serves as a warning of disease or a threat to the body. In contrast, chronic pain is a persistent and debilitating condition for which there are few treatment options. Chronic pain conditions include arthritis-induced pain, cancer pain, chemotherapy-induced pain, diabetic pain, migraine, fibromyalgia, and inflammatory and neuropathic pain [1]. In this review, we will mostly present studies involving animal models of inflammatory (e.g., injection of proinflammatory solutions such as carrageenan and complete Freund's adjuvant) and neuropathic pain (e.g., peripheral nerve injury such as spared nerve injury or chronic constriction injury). Previous reviews have been published with detailed descriptions and limitations of using these animal models to study chronic pain [2–5].

Inflammatory pain and neuropathic pain are characterized by spontaneous and evoked pain. Typical evoked pains include hyperalgesia (increased response to painful stimuli) and allodynia (painful response to normally innocuous stimuli). In particular, mechanical or tactile allodynia is probably the most commonly observed symptom in inflammatory and neuropathic pain animal models. Two major neuronal mechanisms underlie this symptom: central sensitization and disinhibition [6]. Central sensitization denotes a state of hyperexcitability of the neurons of the dorsal horn such that their responsiveness to synaptic inputs is increased and involves the modulation of NMDA and AMPA receptors in spinal neurons. The spinal injection of NMDA directly activates dorsal horn NMDA receptors and results in mechanical allodynia [7]. Disinhibition is characterized by a reduction in the effectiveness of the spinal inhibitory GABA and glycine neurons. Pharmacological blockade of GABA or glycine-mediated spinal inhibition also produces mechanical allodynia [8]. The balance between excitatory and inhibitory influences on spinal neuronal circuits plays a crucial role in maintaining physiological pain response. Inflammation or nerve injury leads to an increase in excitation and/or decrease in inhibition resulting in augmented neuronal excitability, which can manifest as chronic pain.

Current treatments of chronic pain include antidepressants, anticonvulsants, sodium channel blockers, NMDA antagonists, and opioids. However, these drugs only target neuronal pathways or symptoms and are limited by their side effects. For instance, opioids are often accompanied by side effects such as respiratory depression, sedation, nausea, vomiting, constipation, dependence, tolerance, and addiction [9]. Therefore, there is an urgent need for new therapeutic targets. Recently, several studies have highlighted the role of nonneuronal mechanisms, such as immune and glial regulation, in chronic pain. Indeed, it is now widely accepted to consider chronic pain as a neuroimmune disease [10–13]. In particular, nerve injury induces significant activation of glial cells in the spinal cord, and the activated glial cells contribute to central sensitization and disinhibition via proinflammatory mediators [14]. Inhibitors of glial cells are able to attenuate chronic pain [15, 16] and may offer new therapeutic avenues.

Microglia are prominent glial cells in the spinal cord and contribute to chronic pain [17, 18]. As of this writing (29 July 2016), a PubMed search for “Microglia Chronic Pain” retrieves 509 articles, of which ~20% were published within the preceding 12 months. Clearly, microglial cells in chronic pain are a hot topic and fast growing area of research. Naturally, in such diverse and rapidly developing research, we cannot possibly cover all of the work that has been carried over the last two decades and we certainly expect additional progress will have been made by the time this review is published. We apologize to authors whose work we have not discussed.

In this review, we summarize the major signaling pathways involved in microglial activation and chronic pain with an emphasis on caspases (CASPs). In particular, potential microglial mechanisms and therapeutic approaches for the modulation of CASP6 in chronic pain are also described.

## 2. Microglia Signaling and Chronic Pain

**Figure 1:**
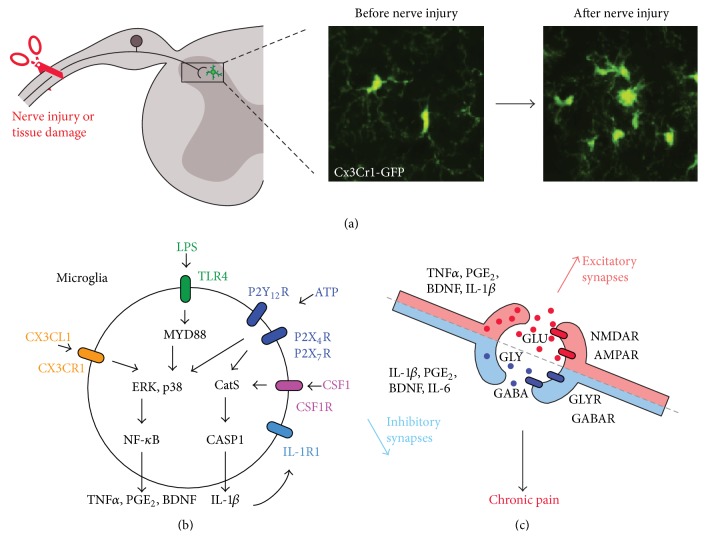
Microglial reactivity to nerve injury and signaling in chronic pain. (a) Nerve injury induces CX3CR1 expression in spinal microglia of mice expressing GFP under the control of CX3CR1 promoter. (b) Tissue and nerve injury results in the release of ATP, CX3CL1, and CSF1 leading to the activation of microglia shown by the phosphorylation of p38 and pERK and the production of prostaglandins, cytokines, and growth factors (e.g., TNF*α* and BDNF). Microglial cells also produce the cytokine IL-1b via the cathepsin/caspase-1 pathway, which can be further activated by binding to IL-1R1. (c) Microglial cytokines, prostaglandins, and growth factors modulate excitatory (glutamatergic synapses—GLU/NMDA and AMPA receptors) and inhibitory (GABAergic and glycinergic synapses—GABA/GABAR and GLY/GLYR) synaptic transmission. For instance, BDNF produces disinhibition of GABAergic lamina I neurons leading to chronic pain.

Microglia are innate immune cells in the central nervous system constantly scavenging their environment using their ramified branches for the maintenance of hemostasis [18]. In animal models with nerve injury, microglia proliferate, change shapes (e.g., larger cell bodies and fewer ramifications), and increase the expression of microglial markers such as CD11b, ionized calcium-binding adapter molecule 1 (IBA1), and CX3C chemokine receptor 1 (CX3CR1) (Figure 1(a)). However, changes in the morphology and expression of these markers are not indicative of microglial activation and participation to pain symptoms. Notably, microglia proliferation and IBA1 expression increased are very limited after tissue inflammation or bone cancer, but pain in these animal models is still efficiently attenuated by the inhibition of microglial signaling [19, 20]. Several studies have reported the microglial phosphorylation of p38 (p-p38) in several animal models of pain involving nerve injury, spinal cord injury, formalin-induced acute inflammatory pain, postoperative pain, and chronic exposure to opioids [21–27]. Furthermore, these studies have also shown that microglial p-p38 leads to the production of proinflammatory cytokines that can alter pain pathways and p38 inhibition significantly attenuates pain. Therefore, the spinal phosphorylation of p38 might represent a better marker for microglial activation and participation in pain compared with classical microglial markers such as IBA1.

Considerable effort has been devoted to understanding the mechanisms by which microglial cells are activated and how they contribute to chronic pain. In recent years, many neuron-microglia pathways have emerged in chronic pain, including the chemokine receptor signaling (e.g., CX3CR1) [28], toll-like receptor signaling (e.g., TLR2 and TLR4) [29, 30], purinergic receptor signaling (e.g., P2Y12R and P2X4R) [31, 32], and tyrosine-protein kinase receptor signaling (e.g., CSF1R) [33] (Figure 1(b)).

Adenosine triphosphate (ATP), chemokines (CX3CL1 and INF*γ*), and proteases (MMP9 and CASP6) are released from the spinal projections of the primary sensory neurons following peripheral tissue or nerve injury. ATP, chemokines, and proteases induce signaling via ligand-gated ion channels and G protein-coupled receptors (GPCRs). These mediators are not unique to primary sensory neurons and may be secondary to microglial activation. For instance, CX3CL1 release requires the production of cathepsin S (CatS, a lysosomal protein), which is induced by the stimulation of the microglial receptors P2X7R or CSF1R [33–35]. Stimulation of the P2X7R also elicits the production of IL-1*β* and its maturation via CatS and CASP1 [36], which can further increase microglia activation via the interleukin receptor IL1R [37] and it is also a key contributor in the hyperexcitability of the nociceptive dorsal horn neurons [14]. IL-1*β* and IL-18 can also be processed by microglial CatB and CASP1/11 in inflammatory pain [38], suggesting a major role for the microglial Cat and CASP signaling in chronic pain.

Probably the most studied and well-characterized microglial signaling pathway is the TLR4 signaling pathway. Toll-like receptors are known to regulate innate immunity and respond to diverse invading pathogens and damage-associated molecular patterns. For example, TLR4 associates lipopolysaccharide (LPS) from the walls of Gram-negative bacteria. TLR4 is predominantly expressed in microglia and spinal injection of LPS-induced pain behaviors [39]. Increased TLR4 expression correlates with the development of pain after nerve injury and its inhibition significantly attenuates nerve injury-induced pain [40–42]. Notably, TLR4- and TLR2-deficient mice demonstrate decreased microglial reactivity and attenuated pain after nerve injury [30, 40]. TLRs have also been proposed to sense endogenous injury signals including fibronectin and heat shock proteins. After nerve injury, HSP90 is upregulated in the spinal cord and its inhibition attenuates TLR4 mediated pain [43].

TLR signaling, such as most of the aforementioned signaling, converges in the phosphorylation/activation of the mitogen-activated protein kinases (MAPK), ERK, and p38 [15]. In particular, p38 is persistently activated exclusively in microglia, whereas ERK is only activated in microglia in the first week after nerve injury [44]. Microglial MAPK phosphorylation usually results in the rapid activation of signal-dependent transcription factors, including members of the nuclear factor-kB (NF-kB) and interferon regulatory factor (IRF) families [23, 45, 46]. These factors can then work in combinatorial manner to rapidly express hundreds of genes known to increase pain sensitivity, including the proinflammatory cytokines TNF*α*, inducible nitric oxide synthase (iNOS), and brain-derived neurotrophic factor (BDNF) as well as the purinergic receptors P2X4R and P2Y12R [19, 47, 48]. In particular, the microglial production of proinflammatory cytokines and neurotrophic factors can further recruit microglia, activate surrounding astrocytes, and promote the sensitization of central nervous system nociceptive circuits (Figure 1(c)).

Proinflammatory cytokines such as TNF*α*, prostaglandin PGE_2_, and IL-1*β* can increase the excitatory synaptic transmission by both pre- and postmechanisms of the excitatory synapses by enhancing the release of glutamate and increasing the trafficking and modulating AMPA and NMDA receptors. In parallel these cytokines, including IL-1*β*, PGE_2_, and IL-6, can also reduce or promote the loss of the inhibitory synaptic transmission (i.e., disinhibition). Furthermore, inhibitory synaptic transmission can also be reversed. Microglial release of growth factor BDNF downregulates the potassium-chloride cotransporter KCC2 in lamina I GABA positive neurons leading to the accumulation of the intracellular chloride, such as these inhibitory neurons changing phenotype and becoming excitatory [49]. Previous reviews have been published and are available with further details about these mechanisms [15, 18, 50].

## 3. Caspase Signaling and Chronic Pain

CASPs are cysteinyl-aspartate-specific proteases and best known for triggering apoptotic cell death [51]. CASPs are generally present in cells as inactive precursor enzymes with little or no proteases activity. Two major pathways regulate the activation of CASPs. The extrinsic pathway is elicited by the biding of extracellular death ligands (such as TNF*α*) to transmembrane death receptors, whereas the intrinsic pathways are induced by cell stress (such as oxidative stress) or damage. Both pathways can lead to apoptosis by the activation of the initiator CASP2, 8, 9, and 10 and the executioner CASP3, 6, and 7, or neuroinflammation via CASP1, 4, 5, and 11 [52].

**Figure 2:**
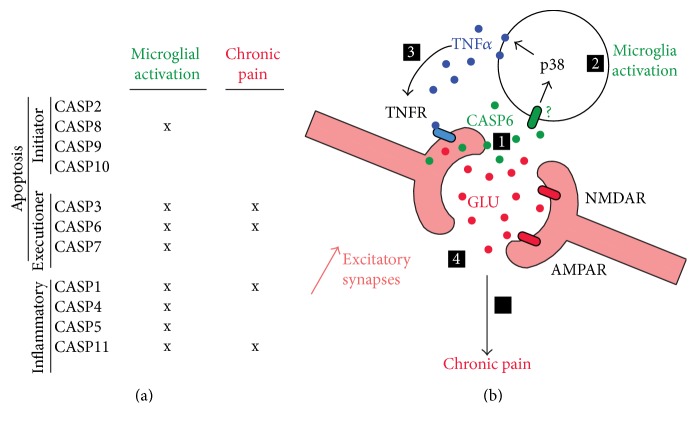
Caspases in microglial activation and chronic pain and schematic representation of the CASP6 neuroinflammatory mechanism. (a) Regulation of apoptotic and inflammatory caspases in microglia activation and chronic pain. (b) Tissue and nerve injury leads to the release of CASP6 from the central afferents of primary sensory neurons [1]; this leads to the microglial activation shown by the phosphorylation of p38 and production of TNF*α* [2]; consequently TNF*α* binds to the presynaptic TNFR increasing the release of glutamate and the excitatory synaptic transmission [3], which ultimately results in chronic pain [4].

Several chronic pain syndromes are associated with increase in TNF*α* and oxidative stress in the both peripheral and central nervous systems, which may lead to CASP activation and neuroinflammation [11]. Indeed several CASPs that regulate microglial activation also participate in chronic pain [19, 36, 38, 53–57] (Figure 2(a)). Although peripheral inhibition of CASP1, 2, 3, 8, and 9 significantly attenuated inflammatory and neuropathic pain behaviors [56], the peripheral mechanisms of CASPs remain elusive. Here, we focus on the central mechanisms of CASP signaling in chronic pain and microglial activation for which we have a better understanding. In chronic pain conditions, CASP activation in the spinal cord leads to both apoptosis and neuroinflammatory responses (e.g., microglia activation).

After peripheral nerve injury, apoptotic cells are observed in the dorsal horn of the spinal cord [57]. This apoptosis is driven by the activation of CASP3 in the inhibitory GABAergic interneurons of the superficial dorsal horn and causes the loss of these neurons, the decrease of the spinal inhibition, and the appearance of neuropathic pain. Spinal injection of pan caspase inhibitor Z-VAD-FMK to block the CASP3 activation prevents the number of apoptotic cells and decrease of spinal inhibition and alleviates neuropathic pain. Interestingly, the alleviation of neuropathic pain by Z-VAD-FMK outlasts its discontinuation, suggesting that degeneration of inhibitory interneurons contributes to the chronicity of pain. However, the neuronal activation of CASP3 and the apoptosis of GABAergic interneurons in animal models of neuropathic pain are controversial [58]. In particular, CASP3 and apoptotic signaling have been shown to occur also in glial cells after the same peripheral injury [58].

Little is known about the potential contribution of CASPs to glial cell functions and chronic pain. However, it has been recently reported that intracranial injection of LPS induces microglial activation of CASP3/7 without leading to apoptosis but instead to the release of proinflammatory mediators and neuroinflammation [55]. As further proof of the central role of CASP3/7 in microglial activation, the use of Z-DEVD-FMK (a specific CASP3 inhibitor) significantly reduced the LPS-induced release of proinflammatory mediators by microglial cells. Interestingly, the spinal delivery of same inhibitor Z-DEVD-FMK or CASP3 siRNA attenuates neuropathic pain after peripheral nerve injury [59]. Whether these treatments attenuate neuropathic pain via the inhibition of apoptosis and/or neuroinflammation remains to be investigated.

CASP1 and CASP11 certainly play a critical role in regulating neuroinflammation and are increased in chronic pain conditions [4, 38]. As briefly mentioned above, the activation of CASP1 and CASP11 plays a role in the maturation of proinflammatory cytokines, such as IL-1*β* and IL-18. The best known activator of CASP1 is the inflammasome, a complex of proteins (such as nucleotide-binding domain leucine-rich repeat containing proteins NLRP1 and NPLR3, NLR family CARD domain-containing protein NLRC4, and the apoptosis-associated speck-like protein containing a CASP recruitment domain or in short ASC) that are aggregated by various inflammatory conditions in immune and glial cells and lead to the CASP1 cleavage [60]. Although several mechanisms can contribute to allodynia after spinal injection of LPS, it has been observed that this treatment enhances CASP1 and ASC secretion providing evidence of the involvement of the inflammasome complex [36]. Furthermore, inhibition of CASP1 by Z-VAD-FMK both prevents IL-1*β* release and attenuates LPS-induced allodynia. However, how and to which extent the inflammasome complex is activated in microglia and chronic pain is still unclear.

There are several variants of inflammasome complexes, but the best know complex involves the activation of NLRP3 [60]. However, a recent study using NLRP3-deficient mice show no defects in regulating the transcriptional expression of ASC, CASP1, and IL-1*β* after spinal injections of LPS or after intraplantar formalin injection or after nerve injury [61]. In a peripheral inflammatory pain model, NLRC4 inflammasome complex but not NLRP3 was implicated in IL-1*β* increase in the skin and behavioral responses [62]. Similarly, NLRC4 and not NLRP3 may be involved in processing spinal IL-1*β* after LPS injection or nerve injury. In contrast, microglial NLRP3/CASP1 seems to be involved in chronic pain arising from the combination of nerve injury and prolonged exposure to opioids [63], suggesting the different pathways and contributions of CASP1 and inflammasome signaling to various neuroinflammatory and chronic pain conditions.

## 4. Caspase-6 in Neuron-Microglia Signaling and Chronic Pain

CASP6 is widely expressed in the brain and in the peripheral nervous system. CASP6 is well known as an executioner CASP and can cleave nuclear structural proteins (e.g., lamin) leading to apoptosis and neurodegeneration. CASP6 is involved in neurodegenerative diseases such as Huntington and Alzheimer diseases [64]. Our recent research suggests that CASP6 has an important and nonapoptotic role in the development of chronic pain in both inflammatory and neuropathic animal models (Berta CASP6).

Several lines of evidence suggest a unique role for this CASP in microglia activation and pain control: (1) CASP6 is highly expressed in the neuronal axons of primary sensory neurons that terminate in the superficial dorsal horn (laminae I-II) of the spinal cord [19, 65], (2) CASP6 is coexpressed with the calcitonin gene related peptide (CGRP), a well-known peptide involved in inflammation and pain [66], (3) CASP6 surrounds microglial cell bodies and processes, and (4) CASP6 levels in the cerebrospinal fluid significantly increase after inflammation [19]. To define the specific role of CASP6 in pain, we employed in vivo and in vitro approaches.

In vivo results in various animal models are very consistent, showing that the CASP6 inhibition by Z-VEID-FMK (a specific CASP6 inhibitor) or deletion can attenuate inflammatory and neuropathic pain (Berta 2014, 2016). Notably, the intrathecal injection of a specific antibody against the active form of CASP6 was effective in blocking formalin-induced pain. Because antibodies with few exception do not penetrate cells [67], this result further proved the presence and role of extracellular CASP6 in pain. In line with an extracellular action of CASP6, intrathecal injection of recombinant CASP6 (rCASP6) is sufficient to induce pain symptoms, such as mechanical allodynia. Importantly, spinal rCASP6 treatment did not produce signs of axonal degeneration, as we found no loss of peptidergic axons (CGRP+) or nonpeptidergic axons labeled with IB4 (an isolectin glycoprotein) in the dorsal horn. However pretreatment with minocycline, a microglial inhibitor that has been shown to attenuate pain [68], significantly reduced the rCASP6-evoked mechanical allodynia, which suggested an action of CASP6 on microglia.

In vitro experiments have demonstrated that stimulation of primary microglial culture with rCASP6 elicited a significant and dose-dependent release of TNF-*α*, but minimal or no release of other proinflammatory cytokines, including IL-6 and IL-1*β* [19]. The treatment of microglia with an inhibitor of p38 suppressed the rCASP6-induced TNF-*α* release, suggesting an important role of p38 in CASP6-triggered TNF-*α* release. Impairment of spinal TNF-*α* was also observed in mice with CASP6 deficiency compared to their wild-type controls after tissue injury and damage [19, 69].

Mechanistically, rCASP6 was also sufficient to enhance spontaneous excitatory postsynaptic currents (sEPSCs frequency) in spinal cord slices via microglial and TNF-*α* signaling [19]. Finally, rCASP6-activated microglial culture medium increased sEPSC frequency in spinal cord slices via TNF-*α*. Together, these data suggest that CASP6 released from axonal terminals regulates microglial TNF-*α* secretion, synaptic plasticity, and chronic pain (Figure 2(b)).

## 5. Conclusions and Future Directions

Millions of people suffer from chronic pain, which is now widely recognized as a neuroimmune disease. However, current treatments are limited to symptomatic palliation mostly focusing on blocking neurotransmission. Targeting neuroinflammation and, in particular, microglial signaling may offer new therapeutic strategies for a better treatment of chronic pain [11].

Spinal microglia contribute to the generation of inflammatory and neuropathic pain, postoperative pain, and opioid-induced tolerance in rodents. Although human studies have established glial activation in chronic pain states using functional magnetic resonance imaging [70] and in postmortem spinal cords [71], clinical trial with inhibitors targeting microglia, such as minocycline (a tetracycline antibiotic) and propentofylline (a CNS glial modulator), has shown no or limited promise in chronic pain [15]. These generic microglia inhibitors are undesirable, since it is well documented that microglia in neuroinflammatory diseases can have multiple phenotypes with pathological and protective functions [18]. Furthermore, nonpathological microglia have an important role within the immune system and are pivotal in maintaining and restoring physiological homeostasis [72]. Therefore, we should target specific pathological microglial signaling.

Among several microglial signaling, CASPs are promising targets to reduce neuroinflammation and chronic pain. Following tissue and nerve injury, CASPs play an important role not only in apoptosis, but also in mounting neuroinflammatory responses including microglia activation. There is increasing evidence that CASP1, CASP6, and CASP11 activation is involved in microglia activation and the maturation of proinflammatory cytokines in inflammatory and neuropathic pain conditions [19, 38, 53, 63]. Inhibition of these caspases has shown extraordinary promise in various disease models including painful conditions. For example, a blockade of CASP6 by the peptide Z-VEID-FMK reduces inflammation and neuropathic-induced mechanical allodynia in mice models [19]. Unfortunately, the vast majority of CASP inhibitors are peptides that often lack selectivity [73].

Our data suggest that CASP6 released from axonal terminals regulates microglial TNF-*α* secretion, synaptic plasticity, and chronic pain (Figure 2(b)). Because of this unique presence of CASP6 in the extracellular milieu, we hypothesized that CASP6 could be targeted by antibodies. Indeed, formalin-induced second-phase pain was suppressed by spinal injection of a neutralizing antibody against the activated form of CASP6 [19]. The use of this antibody can confer many therapeutic advantages over the peptide inhibitor, including selectivity and accessibility. Furthermore, new technologies are available to enable antibodies to cross the blood-brain barrier [74].

It is worth noting that CASP6 inhibition attenuates mechanical allodynia in male, but not in female, mice [69]. Although microglial proliferation occurs similarly in male and female rodents, it has been reported that ablation of microglia and inhibition of microglial signaling attenuate inflammatory and neuropathic pain only in males, but not females [75]. These findings highlight the importance of including both sexes in basic research and they should be considered for future human trials and clinical practice. However, microglia may play different roles in different phases of chronic pain development as well as in different chronic pain conditions. Notably, it has been shown that inhibition of microglial signaling in animal models of bone cancer pain and spinal cord injury effectively attenuated chronic pain also in females [30, 76].

In conclusion, given the pace of recent advances in our appreciation of chronic pain as a neuroimmune disease and in our understanding of the reciprocal signaling between neurons and microglia (i.e. CASP6 signaling), it is at last realistic to expect that new and improved treatments will become available for a more successful management of clinical chronic pain.
